# Monitoring of Single-Cell Responses in the Optic Tectum of Adult Zebrafish with Dextran-Coupled Calcium Dyes Delivered *via* Local Electroporation

**DOI:** 10.1371/journal.pone.0062846

**Published:** 2013-05-07

**Authors:** Vanessa Kassing, Jacob Engelmann, Rafael Kurtz

**Affiliations:** 1 AG Active Sensing and Center of Excellence ‘Cognitive Interaction Technology’, Bielefeld University, Bielefeld, Germany; 2 Department of Neurobiology, Bielefeld University, Bielefeld, Germany; Institut Curie, France

## Abstract

The zebrafish (*Danio rerio*) has become one of the major animal models for *in vivo* examination of sensory and neuronal computation. Similar to *Xenopus* tadpoles neural activity in the optic tectum, the major region controlling visually guided behavior, can be examined in zebrafish larvae by optical imaging. Prerequisites of these approaches are usually the transparency of larvae up to a certain age and the use of two-photon microscopy. This principle of fluorescence excitation was necessary to suppress crosstalk between signals from individual neurons, which is a critical issue when using membrane-permeant dyes. This makes the equipment to study neuronal processing costly and limits the approach to the study of larvae. Thus there is lack of knowledge about the properties of neurons in the optic tectum of adult animals. We established a procedure to circumvent these problems, enabling *in vivo* calcium imaging in the optic tectum of adult zebrafish. Following local application of dextran-coupled dyes single-neuron activity of adult zebrafish can be monitored with conventional widefield microscopy, because dye labeling remains restricted to tens of neurons or less. Among the neurons characterized with our technique we found neurons that were selective for a certain pattern orientation as well as neurons that responded in a direction-selective way to visual motion. These findings are consistent with previous studies and indicate that the functional integrity of neuronal circuits in the optic tectum of adult zebrafish is preserved with our staining technique. Overall, our protocol for *in vivo* calcium imaging provides a useful approach to monitor visual responses of individual neurons in the optic tectum of adult zebrafish even when only widefield microscopy is available. This approach will help to obtain valuable insight into the principles of visual computation in adult vertebrates and thus complement previous work on developing visual circuits.

## Introduction

During the past years the zebrafish (*Danio rerio*) has been established as a valuable model organism to address key questions of neurophysiology and developmental neurobiology by *in vivo* experiments (reviews: [1]–[4]). Major reasons for the increasing popularity of this model organism are its amenability to genetic approaches in combination with behavioral paradigms, and the ability to study the activity of populations of neurons in various regions of the neural system directly by the use of fluorescent calcium indicators.

One brain region that is currently intensively studied in larval zebrafish is the optic tectum, which plays a key role in the transformation of incoming visual signals into task-specific locomotor output and in multisensory integration (review: [5]). Functional *in vivo* imaging along with the ability to genetically target specific types of neurons provides a chance to gain a comprehensive understanding of the cellular computations underlying fundamental sensory-motor functions of the optic tectum [6]–[10].

The optic tectum in teleost fish is, due to its position at the surface of the brain, ideally suited for *in vivo* optical imaging [6]–[8]. Conventionally, imaging is performed in zebrafish larvae up to the age of 15 days post fertilization [11], embedded in a block of low-melting agarose. With later developmental stages, imaging becomes increasingly hampered by skin pigmentation. This problem can, in principle, be avoided by using mutations with pigmentation defects [12], [13] or by treatment with the melanin synthesis inhibitor phenylthiourea [14]. However, besides potential side effects [15], even with these approaches the standard imaging procedures become more difficult with age, because water perfusion through the gills is needed and because the development of the cranial roof limits the optical tissue transparency and the accessibility with dye injection electrodes. Similar constraints exist in the tadpole of *Xenopus laevis,* another important animal model for the study of neuronal processing in the optic tectum [16], [17]. As a consequence, the analysis of the response properties of tectal neurons by optical imaging and the study of their interactions were so far limited to larval stages (day 6–15 post fertilization). Although considerable insight into the principles of maturation of neuronal circuits and into neuronal processing in the immature tectum was gained in these studies [6]–[8], the general relevance of the findings for the functioning of tectal circuitry in adult animals may be limited. To overcome this problem we here present a methodology that allows registering the responses of tectal neurons in adult zebrafish by *in vivo* calcium imaging.

The principal method applied so far to image neural activity in the zebrafish optic tectum builds on the use of membrane-permeant calcium dyes, which offer the advantage that numerous neurons can fairly easily be loaded by pressure ejection of the dye solution into the tissue. This is referred to as “bolus-loading” or “bulk-loading” technique [18], [19]. One disadvantage of this approach is the strong crosstalk of signals from individual neurons, in particular in tissues which are densely packed with neurons. As this is the case in the zebrafish optic tectum, in most studies so far the fluorescence signals from the somata of individual neurons were detected by two-photon microscopy [20]. This method efficiently enhances image resolution even deep inside living tissue by limiting fluorescence excitation to a single focal plane and by using a point-by-point detection scheme that reduces the corruption of image quality resulting from the scattering of emission light. Unfortunately, two-photon microscopy is associated with high costs, which preclude less well-equipped laboratories from the conduction of functional imaging studies in zebrafish. Light field microscopy has been used to enable three-dimensional calcium imaging in larval zebrafish tectum [21], but is still not widely established as an alternative approach to two-photon imaging.

In the present paper we demonstrate that a low-cost approach for calcium imaging in zebrafish optic tectum is feasible, yielding stable single-cell response data from adult animals. Instead of membrane-permeant calcium dyes our approach comprises the use of dextran-coupled dyes, which can be loaded into groups of cells *via* local electroporation or by a mechanical procedure. With our technique, cross-talk between neurons is reduced, because dye staining is restricted to tens of neurons or less, allowing us to resolve somatic calcium signals even with conventional widefield fluorescence microscopy. To prove our method for functional imaging during sensory stimulation we show the responses of single direction-selective and orientation-selective neurons in the optic tectum of adult zebrafish.

## Methods

### Animal Rearing

Adult zebrafish were obtained from a local fish dealer and sheltered in a 100 l aquarium in groups of 5 to 25 fishes. The light-dark cycle was set to 12∶12 hours.

### Animal Preparation and Dissection

All the procedures for animal maintenance and preparations described in this paper comply with the current animal protection law of the Federal Republic of Germany, and were evaluated and approved by the local authorities (LANUV NRW: 87-51-04.2010.A202).

**Figure 1:**
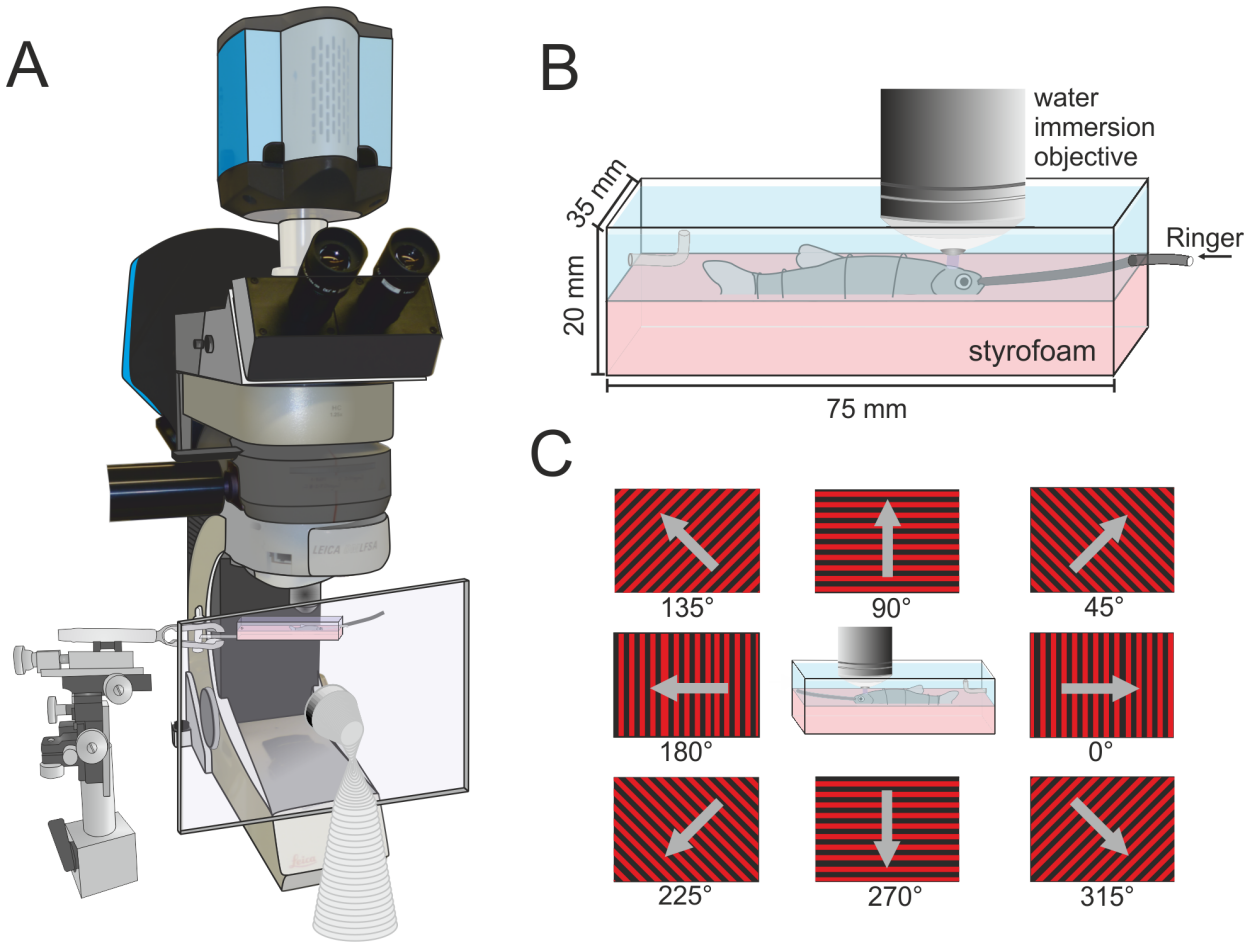
Schematic drawing of the experimental set-up. **A.** The animal was placed under a standard upright fixed-stage microscope, equipped with a sensitive electron-multiplying CCD camera and LED-based epifluorescence illumination. To deliver visual stimuli, we used a TFT screen, which was placed in front of the eye facing the experimenter (drawn transparent for clarity). **B.** Detailed view of the recording chamber. Fish were placed on a styrofoam support tray and held in place by tungsten wires bent to support the animals. The chamber was continuously perfused with Hickmann ringer. **C.** Schematic display of the grating stimulus used for the determination of orientation and directional selectivity of tectal cells. For all data the denomination of the motion directions corresponds to the one shown here.

Zebrafish were anesthetized in a small beaker with 0.05% tricaine methane sulfonate (MS-222, Sigma-Aldrich Chemistry, Steinheim, Germany) and then fixed with pins in a custom-made preparation Perspex chamber (Figure 1B). To fix the fish inside the chamber a piece of styrofoam was attached on the bottom of the chamber. After anesthetizing, the fish was immobilized with an intramuscular injection of 5 µl Pancuronium bromide (1∶100, Braun-Melsungen, Melsungen, Germany) and the gills were irrigated with Ringer solution (Hickmann-Ringer; composition (g/l): NaCl: 7.25, KCl: 0.38, MgSO_4_: 0.11, NaHCO_3_: 1, NaH_2_PO_4_ ·2H_2_O: 0.41, CaCl_2_·2H_2_O: 0.21; pH = 7.2) through the mouth. A drip chamber was used to control the gravity-driven flow velocity. To ensure that the solution passed over the gills, part of a small pipette tip fitting the width of the mouth of the fish was used as an outlet attached to the wall of the chamber. A local anaesthetic (Xylocaine Gel 2%, Astra Zeneca, Wedel, Germany) was applied to the skin above the tectum with a small piece of cotton and left there for a few minutes to have an effect. Afterwards, a small opening (approximately 1.5 mm^2^) was drilled carefully through the cranial roof (Micromot 50E, Proxxon, Föhren, Germany) and the meninges were removed with a forceps to expose the brain. Care was taken not to injure the brain or the blood vessels on top of the tectum. After this procedure the tectal roof was directly accessible.

### Staining Procedures

To fill neurons in the optic tectum with fluorescent calcium dye we pulled micropipettes (Warner G150TF-4 glass tubing, Warner Instruments, Hamden, CT, USA) with a Sutter P-97 puller (Sutter Instruments, San Rafael, CA, USA) to have resistances of 10 to 25 MΩ and filled them with 5% Calcium Green-1 dextran (3000 MW, Molecular Probes, Eugene, OR, USA) in 50 mM HEPES/5 mM KCl in the tip and 1 M KCl in the back end. Under visual control through a stereo microscope, we inserted the microelectrode with a z-stepper micromanipulator (SM-7, Luigs & Neumann, Ratingen, Germany) into the optic tectum at a depth of 20–80 µm. Using an amplifier (VF-1800 High voltage micro-electrode amplifier, Bio-Logic, Claix, France) with a high-gain headstage and a function generator (DG1022, Function/Arbitrary Waveform Generator, Rigol, Oakwood Village, OH, USA) symmetrical current pulses (1 Hz sine wave, 6–10 µA peak-to-peak amplitude) were applied for 1–2 min to label the neurons by electroporation (method modified after [22], [23]). Sine waves were chosen as to minimize the risk of blocking the tip of the electrodes through polarization of the tip [24]. In some animals up to three electroporations were done at different positions of the tectum.

Experiments either commenced one hour after the electroporation, or after an incubation time of approximately 15 hours. In these cases fish were allowed to recover from anaesthesia and swim in the Ringer solution overnight, allowing for a better transport of the dyes. The prolonged incubation time resulted in reduced background fluorescence and enhanced the contrast of single cells.

As an alternative approach to local electroporation we used “pin-injections” to stain neurons with the calcium dye in some experiments. After the same dissection procedure as described above we applied a small droplet of the 5% Calcium Green-1 dextran (MW 3000) solution onto the surface of the tectal roof and inserted an etched tungsten wire a few times into the upper layers of the tectum. With this technique the dye was inserted into the brain via small, mostly reversible injuries of the brain tissue.

### Calcium Imaging

If the dissection procedure was performed the day before the fish was cooled down with cold water and then immobilized with an intramuscular injection of 5 µl Pancuronium bromide. For calcium imaging the fish was fixated with bent wires in the recording chamber (Figure 1A,B) and the gills were irrigated as during the dissection procedure. We recorded relative cytosolic calcium concentration changes by epifluorescence imaging of Calcium Green dextran emission using a water immersion 25× (Leica HCX IRAPO L 25×/0.9 W, Leica Microsystems, Wetzlar, Germany) objective at an upright fixed-stage microscope (Leica DMLFSA, see Figure 1A) equipped with an electron-multiplying charged-coupled device (EMCCD) camera (Andor iXon DV887-BI, Andor Technology, Belfast, Northern Ireland). During image acquisition at a resolution of 512×512 pixels fixed frame rates between 12 to 30 Hz were used in different experiments. Camera control and image acquisition were performed using ImSpector 3.20 (LaVision Biotec, Bielefeld, Germany). 470 nm excitation light was provided by a Leica Fluo LED 4000 light source (filter set: excitation BP 470/40 nm, dichroic mirror 510 nm, emission LP 515 nm and BP 530/50 nm).

### Visual Stimulation

A high-brightness 10.4″ TFT display (F510EK005, Reikotronic, Cologne, Germany; nominal maximal white luminance 1000 cd/m^2^) with a frame rate of 60 Hz was used to present motion of a sine-wave grating or a random-dot pattern in various directions (Figure 1C). Alternatively, counterphase flicker of the sine-wave grating in various orientations or full field flicker was presented with the display. The grating had a temporal frequency of 4 Hz and a spatial wavelength of 10° (for motion and counterphase flicker). The random dot pattern was composed of randomly scattered bright squares with an edge length of 2.8° on a dark background. Approximately 6% of the pixels of the display were bright. The velocity of the random dot pattern was 40°/s. Each stimulus presentation started with a period of 2 s during which the stationary pattern was shown, followed by 4 s of pattern motion or flicker and finished with 4 s of stationary pattern presentation. Only the red channel of the TFT display was activated to avoid cross talk with the detection of fluorescence emission in the green wavelength range. Additionally the screen was covered with a red filter (LP 550 nm), resulting into measured brightness values of 76 cd/m^2^ for the brightest pattern regions and 0.6 cd/m^2^ for the darkest pattern regions. The screen covered ca. 40 degrees in elevation and 50 degrees in azimuth to both sides from a point centered approximately to the optical axis of one eye. Stimuli were designed with self-written programs using OpenGL/Vision Egg [25].

### Data Analysis

Calcium concentration signals were evaluated as changes from the fluorescence baseline level of the Ca^2+^-sensitive dye (ΔF/F_0_ = (F−F_0_)/F_0_; ΔF = change in fluorescence, F_0_ = baseline fluorescence), calculated pixel-wise (for colour-coded images) or within regions of interest (ROIs). For the baseline values (F_0_) we used the average of the images during the first 5–20 frames in the series prior to stimulation. Using the mean responses obtained at each trial and each pattern direction the direction selectivity of neurons was analysed based on the Rayleigh test [26]. To quantify the selectivity for stimulus orientation, we plotted each single recording as a data point in a polar plot, with the mean response during the stimulation interval given as the distance of the data point from the centre in the direction of stimulus motion. We least-mean-square fitted a standard ellipse to these points. As a measure for orientation selectivity we compared the length of the axes of the ellipse. As an orientation selectivity index (OSI) we defined the value 1−(minor axis/major axis). Thus, the OSI can take values between 0 (indicating no orientation selectivity) and 1 (indicating strongest possible orientation selectivity). To test for statistical significance, we used a Monte-Carlo-approach to estimate the distribution of chance level OSI values obtained for the data set of a given recording [27]. As a basic principle of this approach, the recorded data traces were randomly assigned to the different stimulus orientations. Standard ellipses were fitted to each of 10,000 control datasets generated by this random shuffling procedure. As a measure of error probability we then determined how many of the fits to these random datasets produced OSI values higher or equal to the measured values. Based on the same ellipse fits direction selectivity was evaluated: we determined the metric distance of the centre of the ellipse from the centre of the coordinate system and defined a direction selectivity index (DSI) as the ratio of this value to the radius of the major axis of the ellipse (distance to centre/radius of major axis). Similar to the OSI, the DSI is expected to vary between 0 and 1, indicating no and strongest possible direction selectivity, respectively. As for orientation selectivity, a Monte-Carlo-approach was used to evaluate the error probability for the determined direction selectivity. Additionally, statistical significance of direction selectivity was determined by the Rayleigh test. We used Matlab (The Mathworks, Natick, MA, USA) and ImageJ (U. S. National Institutes of Health, Bethesda, MD, USA) for data analysis.

An inherent problem in widefield microscopy is contamination of the signal from nearby out-of-focus structures. We used two precautions to restrict such contamination. First, during an experiment we checked whether further somata were located above or below the chosen ROI and only selected regions where this was not the case for further investigation. Second, we checked for the contamination from out-of-focus regions offline in the following way. We placed nine 4×4 pixel ROIs uniformly distributed along a linear transect through the major axis of the soma under investigation. The inter-ROI spacing was one fourth of the soma diameter with one ROI in the centre of the soma. Using the grating stimulus that evoked the strongest response in the soma, we then obtained the ΔF/F signals of all ROIs. Only somata where the peak signal was clearly centered on the somatic regions and fell off steeply at its borders were included in the current study.

### Histological Preparation

After the experiment the fish was sacrificed by immersion in an overdose of MS-222. The cranial roof was removed and the fish was subsequently fixed overnight in 4% PFA (Paraformaledehyde prills, Sigma-Aldrich). For preparing the brain for cryo-sectioning it was removed from the skull on the next day and placed in an ascending series of 4% PFA-Sucrose solution (10%, 20% and 30% Sucrose). Afterwards the brain was embedded in embedding matrix for frozen sectioning (Thermo Fisher Scientific, Schwerte, Germany), frozen in a conventional freezer for 30 minutes and sliced on a cryostat in 40 µm sections (Reichert-Jung Frigocut 2800, Nussloch, Germany). The brain sections were directly placed on slides, mounted with fluorescence medium (H-1200, Mounting medium for Fluorescence with DAPI, Vectashield, Vector Labs, Burlingame, CA, USA) and coverslipped. Sections were examined using a Leica TCS SP2 confocal system (oil immersion objective HC PL APO CS 20.0×0.70 IMM/COR).

For reconstructing whole stained cells in situ the brain was also removed from the skull and fixed in 4% PFA for at least 24 hours. Then it was placed in the *Scale A* solution [28] for about 2 weeks. Afterwards it was embedded in 1% Agar in a small petri dish and examined via a confocal laser scanning microscope (Leica TCS SP2 confocal system with 40-fold magnification, water immersion objective HCX APO L 40×/0.8 W UVI). The image resolution was 1024×1024 pixels and the line scan speed was 400 Hz, resulting in a pixel dwell time of 2.4 µs and a frame rate of 0.39 Hz. Pixel grey levels were stored with a resolution of 12 bit. The bleached brain can be stored in 4% PFA for prolonged times. We re-imaged a stack after 2 months in this solution without severe loss of signal intensity or tissue stability. Sections were processed using ImageJ (U. S. National Institutes of Health, Bethesda, MD, USA) and BioVis3D software (Dufort y Alvarez 3262, Montevideo, Uruguay).

## Results and Discussion

In teleost fish, the optic tectum is the major target area of retinal ganglion cell axons [29], [30]. Neurons in the optic tectum of zebrafish have been shown to respond selectively to distinct features of the visual input [31], [32]. In zebrafish larvae, a large fraction of tectal neurons are motion sensitive [7]. As shown by ablation studies, locomotor behavior that relies on the processing of visual motion, such as obstacle avoidance and prey capture, is driven by tectal circuits [33], [34]. The response properties of neurons in the optic tectum in adult zebrafish have not been studied as extensively as in zebrafish larvae, with the exception of an older series of extracellular recording studies [31], [32], [35]. In these studies, as well as in similar studies on goldfish [36]–[38], a subset of the neurons in adult optic tectum was shown to possess fairly large receptive fields and to respond in a direction-selective way to moving stimuli [31]. Unfortunately, neural activity monitoring by calcium imaging, which has been applied in almost transparent zebrafish larvae with large success [6]–[8], is not easily applicable in adult zebrafish. The strong pigmentation and the bony cranial roof of adult zebrafish require more demanding preparation techniques than in larvae. Moreover, whereas fish larvae can be kept in a block of low-melting agarose during the entire experiment, when working with adult fish the skull has to be surgically opened and water perfusion through the gills is required all the time.

### Staining of Neurons in Adult Zebrafish Optic Tectum with Dextran-coupled Calcium Dye and Investigation of Neuronal Morphology by Histological Procedures

We aimed to develop a method to stain small groups of cells in the optic tectum of adult zebrafish with a calcium-sensitive fluorescent dye for the registration of visually driven activity of individual neurons. We used local electroporation of Calcium Green-1 dextran (MW 3000, Molecular Probes, Life technologies, Darmstadt, Germany). For this, following craniotomy, we inserted a glass electrode (10–25 MOhm resistance) into the optic tectum and ejected the dye from the electrode tip locally into the tissue by current injections. Different protocols were tested for the current injection, aimed to induce dye uptake by the cells in the vicinity of the electrode tip only. In most of our experiments we used a protocol with sinusoidal current injections (see Methods for details), but successful staining was also obtained with protocols using brief rectangular current pulses. As an alternative procedure, we applied a droplet of saline containing the calcium dye onto the surface of the tectum. A tungsten wire with a tip that was etched to a small diameter was then inserted into the upper layers of the tectum. This procedure is similar to tract-tracing techniques, where crystalline tracers are incorporated into neurons by local and reversible disruption of their membranes [39]. After preliminary experiments to optimize the parameters of each of the two different procedures, we achieved successful staining of neurons with either procedure in more than 80% of our experiments. Among these, one or more visually responsive cells (see following section) were found in half of the preparations (12 out of 24 stainings). Successful staining of small groups or individual neurons with calcium dyes by electroporation has previously been achieved in brain slice preparations and in *in vivo* experiments of the rat neocortex [40], in the brain of an insect, the silkmoth (*Bombyx mori*) [22] and in *Xenopus* tadpoles [16]. In the latter study, staining of single neurons in the optic tectum with Oregon-Green-Bapta-1 was achieved either by electroporation or by current injection during brief whole-cell patch-clamp recordings. Thus our approach is complementary to these studies as it yields efficient labeling of individual neurons in the brain of adult fish, without the requirement of single-cell electrical recording.

**Figure 2:**
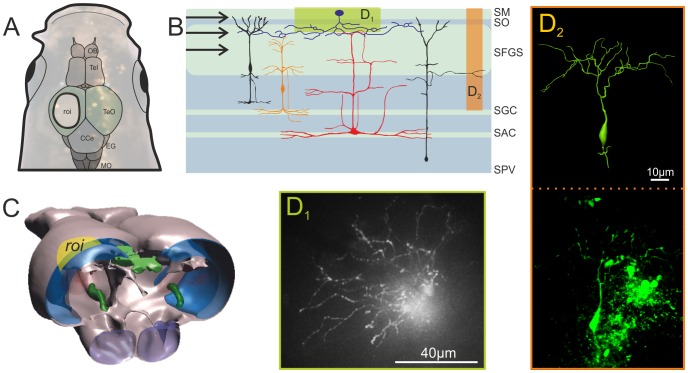
Morphology of neurons in the optic tectum of adult zebrafish. **A.** Image of the head of an adult zebrafish following exposure of the left tectum opticum. A schematic drawing of the brain is superimposed on the image of the head of a zebrafish. The region exposed for imaging is marked as the region of interest (ROI). **B.** Schematic transversal section through the tectum opticum (redrawn after [30]) showing the input layer and the overall cytoarchitecture of some prominent neurons. Areas highlighted in colour indicate where photomicrographs of labelled cells shown in **D_1_–D_2_** are located. **C.** Schematic three-dimensional view on the tectum. The ROI is highlighted in yellow, while the tectal areas are indicated in blue (BioVis3D). **D_1_–D_2_.** Photomicrographs of stained cells. **D_1_** shows an example of a top view using the in vivo imaging set-up (widefield fluorescence). **D_2_** is an example of a neuron recovered after the in vivo experiments following routine histological methods. Here a confocal stack of the vibratome sectioned tectum was used to reconstruct the 3D properties of this neuron (BioVis3D). Abbreviations: SM Stratum moleculare; SO Stratum opticum; SFGS Stratum griseum et album superficial; SGC Stratum griseum central; SAC Stratum album central; SPV Stratum periventriculare; OB Olfactory bulb; Tel Telencephalon; TeO Tectum opticum; CCe Corpus Cerebellare; EG Eminentia granularis; MO Medulla oblongata.

With our approach developmental stages beyond the early unpigmented stages (day 9 post fertilization), can be investigated with conventional widefield calcium-imaging techniques, as described in detail in the next section. Of further benefit for functional imaging studies is the ability to reconstruct and trace labelled and physiologically investigated single cells following the calcium-imaging approach. This ability is an advantage over extracellular recording studies, in which morphological characteristics of the recorded cells remain unknown, often even leaving uncertain whether the recorded signals originate from the terminals of retinal ganglion cells or from tectal neurons themselves [32], [41], [42]. The dextran-based labelling allows for fluorescence microscopy as well as additional immune-histological staining approaches after physiological data of identified neurons has been obtained (Figure 2). Thus one can combine the three virtues of biotinilated dextran amine conjugate (BDA) based fluorescent labeling in a single preparation, namely the ability to measure calcium transients in neurons (see next section), the retrograde transport of BDA for targeting specific neurons and its amenability for routine histological procedures. The latter include immune-histochemistry and electron-microscopy of previously investigated neurons, which can be obtained through photo-conversion [43] or through a standard avidin-biotinylated HRP (ABC) procedure [44], [45].

To prove this, we treated brains following the calcium imaging procedure for both cryo- and vibratome histological procedures. These approaches resulted in brain tissue specimen, in which neurons were labelled sufficiently well to trace and reconstruct their morphology (Figure 2D_2_). Of particular interest in this respect is the recently developed technique of clearing neuronal tissue to enhance the resolution and penetrating depth of fluorescent microscopic approaches [28]. The protocol, based on immersion of the tissue in urea, was shown to enable unprecedented resolution and penetration depth using genetically encoded fluorescent markers in the mouse brain. We have successfully adopted this approach using adult zebrafish tissue, showing that our Calcium-Green marker sustains this treatment (see movie S1).

### Visual Response Properties of Neurons in Adult Zebrafish Optic Tectum

**Figure 3:**
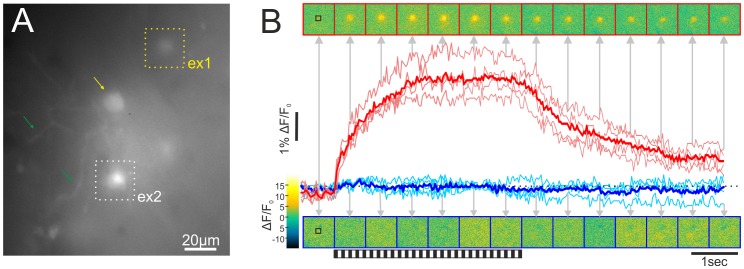
Recording of somatic calcium signals in the optic tectum of adult zebrafish. **A.** Top view on the exposed tectum, showing several somata (yellow arrow) and some dendrites (green arrows). Note that not all aspects of the labelled neurons are in focus, since this is the view based on the experimental focus on the soma indicated by the white dotted square (ex2). Another soma, depicted by the yellow dotted square (ex1), yielded the calcium responses shown in panel B. **B.** Example of the responses to the preferred (45°, red trace) and a non-preferred (180°, blue trace) motion direction of the soma shown in A (ex1, yellow dotted square). Pink and light blue traces show the single repetitions (4 per direction) and the red and dark blue traces show the mean. Single colour-coded images showing the time course of the fluorescence change are depicted at the times indicated by the grey arrows. The first image shows the fluorescence before the pattern starts to move. Every following picture shows the fluorescence in steps of 20 frames. Stimuli lasted four seconds and the stimulus start and end are depicted by the striped pattern below the images. The time courses show the relative fluorescence changes (ΔF/F_0_) averaged over the pixels within a rectangular ROI (indicated as black square in the first image of each series). Baseline fluorescence (F_0_) was determined by averaging across the first 5–20 recorded frames of the series.

Throughout our calcium imaging experiments we used conventional widefield microscopy to visualize the stained neurons, in contrast to other groups, who used two-photon microscopy for imaging of tectal neurons in zebrafish larvae [7], [8], [11]. In another study labelling of particular cell classes with genetically encoded calcium sensors was used [6]. In this study, a microscope with a special semi-confocal principle (the “Zeiss LIVE”, which uses a slit instead of the confocal pinhole) was used, and population calcium signals below single-cell resolution were evaluated in most of the experiments. However, single-cell resolution was reached in this study when using a genetic mosaic expression approach to restrict dye staining to single neurons. Overall, the staining patterns obtained with these approaches are different from those of the local dye application procedures of the present paper, which stain small groups of neurons (Figure 3A), in the range of 5–25 cells, as estimated by the number of visible somata. The contrast of these somata against the background was limited due to the use of widefield microscopy and because calcium-green dextran is present in the extracellular space, in particular close to the injection sites. This is different when dye-filling during intracellular single-cell recording is used, as well as when membrane-permeant AM dyes are used, which only become fluorescent after esterase cleavage within the cells. Nevertheless, usually at least some of the stained somata were clearly visible and sufficiently separated from one another to allow registration of their fluorescence signals without excessive crosstalk. Additionally, incubation times of up to 15 hours between the injection of the dye and the experiment greatly reduced background fluorescence.

To assess the visual response properties of neurons in adult zebrafish tectum we compared the calcium signals obtained during presentation of different types of stimuli. In particular, we asked whether these neurons respond specifically to visual motion in a direction-selective manner or whether particular orientations are preferred. To this end, responses to motion of a sinusoidal grating in various directions (as depicted in Figure 1C) were compared with the responses to counter-phase flicker, i.e., stationary brightness reversals, of the same pattern. The temporal frequency was the same for motion and flicker to induce local brightness modulations with identical temporal profiles for the two conditions. The exemplary cell shown in Figure 3 (ex1) responds differently to motion in different directions. Motion in a direction of 45° (preferred direction), denoting oblique motion from frontal bottom to rear top, induces a strong increase of the fluorescence signal, indicating an increase in somatic calcium concentration, which likely results from an increase in action potential frequency (Figure 3B, red trace). In contrast, pattern motion in a back-to-front direction (180°) does not induce any obvious change in the fluorescence signal (blue trace). This finding demonstrates that neuronal functionality is preserved during our preparation and staining procedures. It further indicates that the examined neuron responds directionally selective to visual motion, as previously demonstrated to be the case for subsets of neurons in the tectum of larval zebrafish [7].

**Figure 4:**
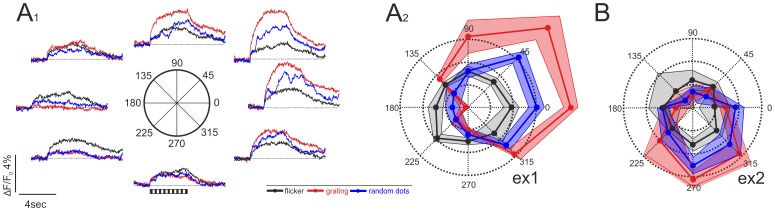
Responses of tectal neurons to three different types of visual stimuli. **A_1_.** Mean responses (n = 3) to grating motion (red), motion of a random dot pattern (blue) and counterphase flicker of the grating (black) of the neuron shown in Figure 3 (ex1) in eight different directions (0°–315°). **A_2_**. Polar plots for the three different types of visual stimuli shown in A_1_. The plot shows the mean amplitude of the responses with the shaded areas indicating one standard deviation. The neuron had a clear and significant directional tuning for both, the moving random dot pattern (blue) and the moving grating (red), but was unselective for the orientation of the flicker stimulus. Throughout the figure, responses to grating motion are shown in red, responses to motion of the random dot pattern are shown in blue, and responses to counter-phase flicker are indicated in black. Note that for uniformity with the motion stimuli each of the four different flicker conditions is represented by pairs of opposite “directions”, e.g. 0° and 180° both denote flicker of a grating with vertically oriented bars (compare Figure 1C). **B**. Example from a second neuron in the same preparation (see white square in Figure 3A, ex2). As in A_1_–A_2_, this cell was directionally selective but with a different preferred direction (290°) and was non-selective for the orientation of the flicker stimulus.

Directional tuning of the neuron shown in Figure 3 was investigated in more detail by presenting stimuli of eight different motion directions repeatedly (n = 4) (Figure 4A_1_). Strong somatic calcium signals during motion in directions ranging from 315°–90° were opposed to very weak responses to motion in directions ranging from 135°–270°. Direction-selective responses were also obtained during motion of a random-dot pattern (Figure 4A_1_, blue traces). Strong response fluctuations, resulting from the coarse texture elements present in the random-dot pattern, were present, and the overall response amplitude remained mostly below that obtained with motion of the sinusoidal grating (Figure 4A_1_, compare red and blue traces). This finding suggests that the neuron does not show a preference for motion of distinct, object-like textures, which are present in the random-dot stimulus but not in the sinusoidal grating. In contrast to the motion stimuli, counter-phase flicker elicited moderate calcium responses in all stimulus conditions (Figure 4A_1_, black traces). For counter-phase flicker, no clear orientation preference for the grating was visible in the mean traces. Moreover, motion in preferred or close-to-preferred direction can drive the neuron to a much higher response level than flicker of any orientation, indicating the presence of circuits that efficiently extract motion cues from the visual input. A polar plot of the responses (Figure 4A_2_) for the different types of stimuli, averaged over the whole stimulus period, illustrates the strong direction selectivity and the similarity of the preferred directions for the two types of motion responses, as opposed to the non-selective character of the flicker responses. Direction selectivity was significant for grating motion (Rayleigh test, p = 0.0002) and for random-dot motion (p = 0.007), but uniform for counter-phase flicker (p = 0.95). Similar results were obtained for another neuron in the same preparation (Figure 4B). Again, direction-selective responses were obtained with motion of the sinusoidal grating (p = 0.002) as well as the random-dot pattern (p = 0.019). However, in contrast to the first neuron preferring motion between 0° and 45° the preferred direction of motion of this neuron was between 270° and 315° for gratings as well as random dots, corresponding to motion from frontal top to rear bottom.

**Figure 5:**
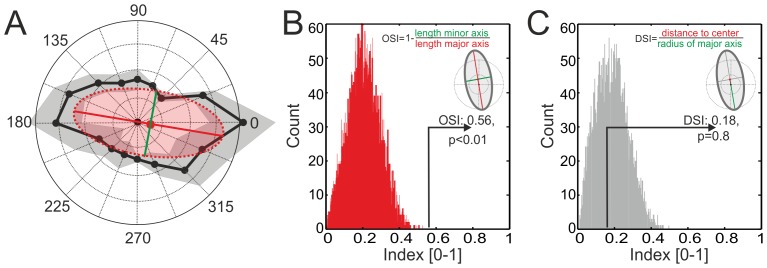
Example of an orientation-selective neuron. **A**. The polar plot in A shows the mean response (black dots) in response to the moving gratings (n = 3) with the standard deviation being shown by the grey shaded area. The neuron was selective for motion in almost horizontal directions (e.g., moving gratings with vertically oriented stripes). The red dotted ellipse shows the fit to the raw data with the major and minor axis of the ellipse depicted by the red and green line. Note that the centre of the ellipse (red dot) is only shifted slightly off origin (black dot), which indicates very weak direction selectivity at most. In contrast, the major and minor axes differ markedly in their length, indicating a strong orientation preference of this cell. Direction selectivity was quantified by the DSI, defined as the metric distance of the centre of the ellipse from the centre of the coordinate system, relative to the radius of the major axis of the ellipse (see Methods and inset in **C**). Orientation selectivity was quantified by the OSI = 1−(minor axis/major axis) (see **A** and inset in **B**). **B, C**. A Monte-Carlo approach was applied to test the robustness of the calculated OSI and DSI values. In each case the distribution of the indices obtained for 10.000 repetitions is shown, with the experimentally determined OSI and DSI values being superimposed by solid lines. Note that orientation preference was highly significant, whereas no directional preference was present. Rayleigh statistics confirmed the lack of directionality tuning of this neuron.

Some of the visually responsive neurons in the adult zebrafish optic tectum were not selective for a particular motion direction, but preferred a certain orientation of the grating pattern, responding with similar strength to opposite directions of motion. An example is shown in Figure 5. We evaluated the orientation selectivity of this neuron by fitting an ellipse to the data points for individual response trials for the various motion directions. The OSI (see Methods) was 0.56 in the example shown. As determined by a Monte-Carlo approach (see Methods), this OSI was highly significant (Figure 5B, p<0.01), whereas direction selectivity was not significant (Figure 5C).

**Figure 6:**
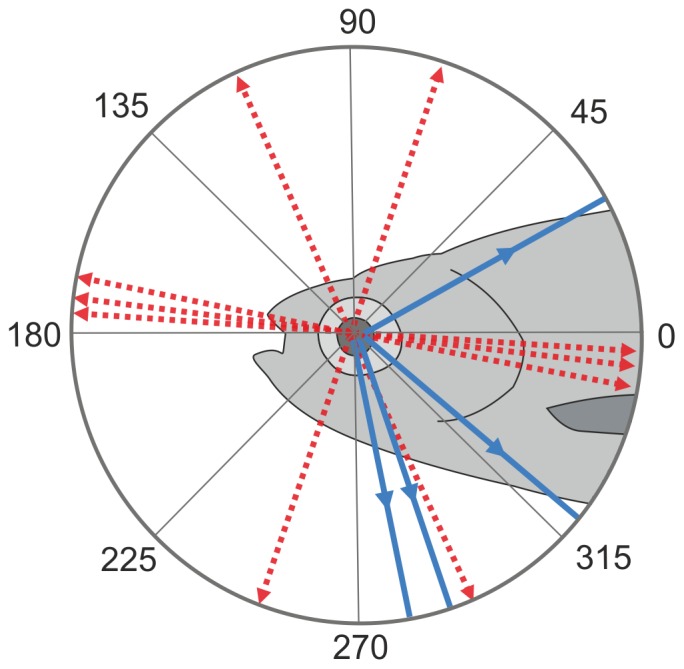
Summary plot of direction and orientation preferences. The blue, solid lines with arrowheads indicate the preferred direction of motion of the significantly direction-selective neurons relative to the fish’s body axis. The red, broken lines with arrowheads at both ends indicate the preferred axes of motion of the significantly orientation-selective neurons. Note that the corresponding stripe orientation of the grating is in all cases perpendicular to these axes (see inset at 45°).

On the whole, we characterized the visual response properties of 23 neurons in 11 fish by evaluating their somatic calcium signals. Out of these, 4 neurons were directionally selective (Raleigh test, p<0.05) and 5 neurons were orientation selective (Monte-Carlo method, p<0.05). In Figure 6 the preferred directions of the directionally-selective neurons and the preferred axes of motion of the orientation-selective neurons relative to the fish’s body axis are summarized. Fourteen neurons were clearly responsive to the applied visual stimuli, but were neither significantly tuned for particular motion directions, nor for grating orientations. However, out of these 14 neurons clear tendencies for directional selectivity or orientation selectivity were observed in 2 neurons in each case, but statistical significance was not reached, primarily due to an insufficient number of stimulus repetitions. In zebrafish, direction selectivity of neurons in the optic tectum has previously been documented by extracellular recording in adult animals [31], [32] and by calcium imaging in larvae [7], [8]. At 9 days post-fertilization nearly half of the neurons responded to a moving dot in a direction-selective way, as defined by a twofold larger response to the preferred than to the opposite direction of motion [7]. A direct comparison with our data is problematic, because the types of motion stimuli and the criteria for defining direction selectivity were different, and because the types of neurons that were examined might be different. Nevertheless, these findings suggest that the proportion of direction-selective neurons is already high in larvae and not much enhanced with further maturation to adulthood.

As known from electrophysiological studies in goldfish [37], [41], [42] as well as from a recent calcium imaging studies in zebrafish larvae [46], [47], inputs to the optic tectum provided by retinal ganglion cells are already selective for a particular axis or a particular direction of motion. Thus, these parameters are not necessarily computed *de novo* in the optic tectum. Nevertheless, there is evidence for further processing steps in the optic tectum, either to enhance direction selectivity [8], [35], [48], or to increase receptive field size [32], or to compute additional stimulus parameters, such as selectivity for small-sized objects [6]. In retinal ganglion cells of zebrafish larvae [46], [47] as well as goldfish [37], [41], [42] the distribution of preferred directions and preferred motion axes of direction-selective and orientation-selective units, respectively, was found to be non-uniform. In both species a large majority of the direction-selective units had a preference for tail-to-head motion. The low number of tectal neurons recorded in our study makes a comparison of their properties with those of the retinal ganglion cells problematic. Nevertheless, it is noticeable that, although a tail-to-head preferred direction is not present among the direction-selective neurons recorded in our study, the clustering of some of the preferred orientation axes indicates that a preference for motion along the horizontal axis might be prevalent among the orientation-selective tectal neurons (Figure 6). Most recently, orientation-selectivity of tectal neurons was reported in a calcium imaging study on zebrafish larvae [49]. Whereas the neurons characterized in this study preferred motion along the vertical axis, the preferred axes of the neurons in our study are more variable (Figure 6). However, the low number of neurons characterized in both studies precludes a systematic comparison of orientation selectivity in larval and adult zebrafish tectum.

**Figure 7:**
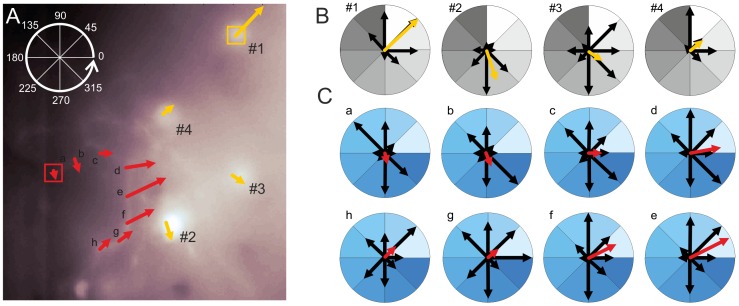
Superficial dendritic structures are accessible and yield consistent Ca^2+^ signals. **A.** Mean raw fluorescence image showing a somatic ROIs (yellow) and a dendritic ROIs (red). The mean orientation vectors obtained at each ROI (sizing 12×12 pixels) are shown centred on the ROIs. **B–C.** Responses to the eight different pattern directions are shown for somatic (B) and dendritic (C) signals. Black arrows represent the normalized response strength for the individual grating orientations. For a given ROI the maximal amplitude was used to normalize the vector lengths. The mean vectors (coloured arrows) are superimposed on these data. Mean vectors are normalized to the strongest orientation tuning found in the somata (ROI 1) and the dendrites (ROI d), respectively.

In a subset of our experiments dendritic structures of tectal neurons could be resolved in addition to their somata. These structures were located in a superficial layer of the optic tectum (Figure 2D_1_ and Figure 7A). Although we did not explicitly focus on dendritic signals in this pilot study, the staining quality (Figure 2D_1_) indicates that it is possible to resolve subcellular calcium signals in these neurons even with conventional widefield fluorescence microscopy. While the neuron shown in Figure 2D_1_ did not give calcium signals in response to visual stimulation, the preparation shown in Figure 3A showed dendritic calcium signals in addition to the somatic signals already illustrated before. In Figure 7 Ca^2+^ signals and their direction tuning are compared within different ROIs covering four somata as well as eight dendritic regions. Note that, although the preparation is the same as shown in Figure 3, the focal plane is slightly different. Faint dendrites are visible, which presumably connect to the soma in ROI #4, located in a slightly deeper layer than the dendrites. The eight dendritic ROIs a-h (Figure 7A,C) show a consistent tuning of the Ca^2+^ signals. Although dendritic signals for single motion directions are fairly variable within one ROI and between different ROIs, the resulting vector for the ROIs appears to shift gradually in a systematic manner along the dendrite. Note that, although the dendritic signals are often weaker than the somatic signals, it is unlikely that they result only from crosstalk of the optical signals, because they differ from nearby somatic signals in their directional tuning (compare e.g. dendritic ROI c and the nearby ROI #2). In the data recorded so far, it was difficult to distinguish exactly which of the processes belonged to one neuron, and to which soma they are connected. Nevertheless, based on this preliminary data we expect that it will be feasible to systematically examine dendritic responses for superficial layers of the tectum based on our approach. Using two-photon imaging it recently was shown for the optic tectum of *Xenopus* larvae that single neurons receive retinotopically arranged local input at their dendrites [16]. Based on our data we expect that it will be possible to investigate dendritic computations underlying direction and orientation tuning in the tectum of adult zebrafish even with widefield techniques. A combination of our staining technique with two-photon imaging certainly will enable similar studies including deeper layers of the tectum. Essential questions that shall be addressed in such studies are how local directional inputs are integrated at the dendrites [50], [51] and whether dendritic processing contributes to the computation of selectivity for certain stimulus features, such as motion direction [52], [53] or object size [6], [54].

### Conclusions

Due to its excellent amenability to genetic tools and physiological techniques, the zebrafish has emerged as one of the most important model animals for the study of neuronal processing. However, the large knowledge about the development of the brain areas that are relevant for sensory-motor computation, such as the optic tectum, contrasts with a lack of knowledge about how sensory information is processed in these areas in adult animals. In the present study we demonstrated that neuronal activity monitoring by calcium imaging, which developed during the last decade as one of the most essential tools to study neuronal processing in larval zebrafish, is also feasible in the optic tectum of adult zebrafish. This offers the opportunity to study in detail the impact of early visual stimulation on the formation of the adult optic tectum. Moreover, studies in larvae are not practical in several other fish species, some of which have since long been used as valuable animal models to analyse specific types of sensory processing, such as cerebellar involvement in electroreception of weakly electric fish [55]. The experimental procedures developed in our study might in the future enable *in vivo* optical imaging of neural activity in these fish species. By the use of electroporation techniques we managed to restrict fluorescence staining to a low number of tectal neurons, thus facilitating the collection of data from individual neurons with widefield microscopy. Whereas studies in larvae focused on monitoring somatic calcium signals in large populations of neurons our approach allows characterization of single-cell response properties and may even open the possibility to compare signals across subcellular structures. Future studies combining the virtues of transgenic lines expressing calcium indicators with our approach on imaging in adult fish might therefore allow to target the principles of retinotopic input integration and dendritic computation within single neurons in the adult zebrafish optic tectum.

## Supporting Information

Movie S1 **Staining of small populations of neurons in the tectum of adult zebrafish with dextran-coupled calcium dye can be used for whole-mount studies following** ***in vivo*** **imaging.** After an experiment, the brain is fixed and removed from the scull. Tissue clearing based on the Scale protocol [28] for 10 days yields a transparent specimen. After clearing the tissue, the neurons that have been stained and for which functional data was obtained can be imaged for in-situ reconstructions (Leica TCS SP2 confocal system).
(WMV)
